# Prevalence of Coccidia and Other Intestinal Parasites in Indigenous Sheep (*Ovis aries*) in an Agricultural Area in Central Nepal

**DOI:** 10.1155/vmi/1033918

**Published:** 2025-06-20

**Authors:** Roshan Babu Adhikari, Madhuri Adhikari Dhakal, Tirth Raj Ghimire

**Affiliations:** ^1^Department of Zoology, Nepalese Army Institute of Health Sciences (NAIHS), Kathmandu 00977, Nepal; ^2^Department of Zoology, Alka Health Institute Pvt. Ltd., Lalitpur 00977, Nepal; ^3^Wildlife Biology, Third Pole Conservancy (TPC), Bhaktapur 00977, Nepal; ^4^Department of Microbiology and Research and Development, New Edge Microbials, Albury 0061, New South Wales, Australia; ^5^Department of Zoology, Tri-Chandra Multiple Campus, Tribhuvan University, Kathmandu 00977, Nepal; ^6^Animal Research Laboratory, Faculty of Science, Nepal Academy of Science and Technology, Lalitpur 00977, Nepal

**Keywords:** *Cryptosporidium*, *Eimeria*, *Fasciola*, fecal consistency, polyparasitism

## Abstract

**Introduction:** Sheep, the multifaceted small ruminants, are vital for meat, milk, wool, manure, skins, and transportation. However, various factors often threaten their sustainability, particularly in lowland areas. Notably, diseases caused by intestinal parasites, particularly coccidian and other helminths, highlight the crucial need for strategic health management in sheep farming.

**Aims:** This study aimed to assess the prevalence and diversity of coccidian and other intestinal parasites in indigenous sheep reared in smallholder farms in the lowlands of Nepal.

**Methods:** A total of 160 fresh fecal samples with age and sex variants were collected via noninvasive techniques. These samples were macroscopically inspected for fecal consistency and transferred to the research laboratory for microscopic examination.

**Results:** It showed a 96.3% prevalence and 26 diverse species of intestinal parasites involving coccidia (84.4%; 12 species), other protozoa (65.6%; 4 species), and helminths (78.1%; 10 species). The prevalence of protozoa (94.4%) was higher than that of helminths (78.1%). Compared to other groups, adults (100%) and female sheep (96.6%) had a higher prevalence rate of intestinal parasites. Additionally, concomitant infection (92.5%) was more common than monoparasitism (3.6%). Notably, sheep with grazing opportunities, thin and weak musculature, mixed domestication with livestock, unknown deworming history, and resting on mud showed higher positive cases.

**Conclusions:** The indigenous sheep in central Nepal are significantly affected by a high prevalence and a wide variety of coccidian and other intestinal parasites. Many of these parasites are associated with severe health conditions and can lead to the death of sheep. Therefore, implementing strategic medication and training programs on healthy rearing practices for local farmers is of utmost importance.

## 1. Introduction

Sheep (*Ovis aries*), Order Artiodactyla, are domesticated small ruminants of significant agricultural and economic value. They are raised primarily for their wool, meat, pelts, dairy products, manure, and even sometimes transportation [1]. They have also been a vital component of human civilization for centuries and served as a popular sacrificed animal in both traditional and contemporary religious rituals in Nepal [2]. At present, 771,205 heads of sheep belonging to four indigenous breeds (purebred), Lampuchhre (12%), Kage (21%), Baruwal (63%), and Bhyanglung (4%), and commercial crossbreed (5%) exist in Nepal [3, 4]. Almost 84% of the major sheep farms are concentrated in the hilly and mountainous regions, and only a few sheep populations (14%) are reared in the Terai plains [5].

In developing and agricultural countries like Nepal, the livestock industry, particularly the sheep industry, plays a substantial role in uplifting the national economy and is an essential source of food, income, and employment for rural farmers [6]. Despite these benefits, sheep farming has not been able to ensure animal welfare and has faced sustainability issues in Nepal. Recent government data shows a 4% decline in the contribution of the livestock sector to the national GDP and a 5% decrease in the existing sheep population over the last 10 fiscal years [3]. This decline is a result of several impediments, like the poor performance of local breeds, seasonal deficits in pasture and other feed, a lack of organized market for wool and meat, including the decreasing market value of wool, poor access to agricultural credit, primitive shearing equipment, inadequate supply of drinking water, better job opportunities, the low interest of the new generation, and diseases such as contagious ecthyma, pneumonia, scabies, Peste des petits ruminants (PPR), and foot and mouth disease [6–11].

While viral, bacterial, and fungal diseases are critical in sheep, gastrointestinal (GI) diseases caused by GI parasites are also challenging threats to these animals. For example, sheep are well known to harbor single-celled protozoa, primarily *Entamoeba* sp. and coccidian, including *Cryptosporidium* sp. and *Eimeria* spp. [12, 13]. Similarly, different helminths, like liver flukes, rumen flukes, tapeworms, roundworms, and strongyles, have been detected in sheep from different geographies [14–16]. Although various factors, like the nature of the infecting parasite, host immunity, environmental conditions, and the intensity of the infection, determine the disease outcomes as explained by the disease triangle [17], the primary parasitic impact includes nutrient deprivation and malabsorption, leading to poor growth, weight loss, and reduced productivity, anemia, diarrhea, and dehydration, immunodeficiency and increased susceptibility to bacterial or viral pathogens, and hypoproteinemia resulting to submandibular edema in sheep [18–20]. Additionally, several cases of mortality of sheep due to heavy parasitic infection have also been reported in different agricultural regions globally [21–24].

To date, only two studies concerning the assessment of intestinal parasites in sheep have been locally conducted in Nepal. These studies with limited sample sizes (*N* = 110–150) have recorded only *Entamoeba* sp. *Eimeria* sp., Strongyle sp., *Trichuris* sp., *Nematodirus* sp., *Fasciola* sp., *Paramphistomum* sp., and *Moniezia* sp. within two different geographical locations in Nepal [11, 25]. However, overall GI parasitic richness in these hosts is yet to be fully illustrated, with prime emphasis on the diversity of coccidian and helminth species, which could significantly impact the survival of sheep [26, 27]. In the same way, the effect of different epidemiological variables on the frequency of parasites in sheep needs further discussion and clarification. Therefore, the current study aims to investigate the prevalence and abundance of intestinal parasites, particularly the coccidia, and helminths, and explain how these pathogens are distributed among sheep in regard to different risk factors, including ages, sexes, rearing and medication practices, and others in an agricultural region in the low land of Nepal.

## 2. Methodology

### 2.1. Study Area

**Figure 1:**
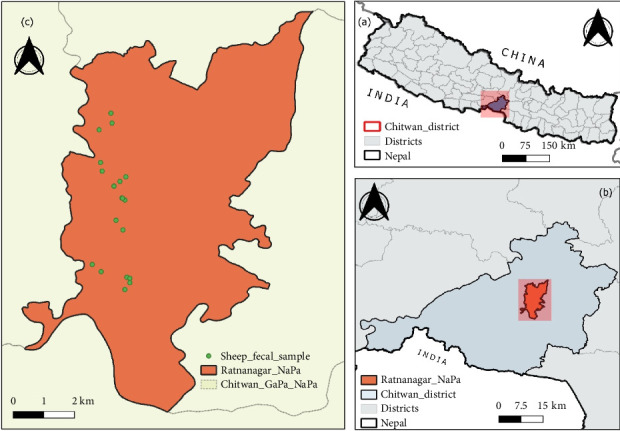
Map of the study area.

This study was conducted within the agricultural regions in Ratnanagar Municipality (27°37′N, 84°30′E) in the Chitwan district, situated in the inner Terai in central Nepal (Figure 1). Ratnanagar has a humid subtropical climate influenced by the monsoon and temperature ranges 25–38°C in summer and 7–20°C in winter, with an average annual rainfall of 2200 mm. The area is rich in fertile agricultural land, and the primary occupations of the people include agriculture, animal husbandry, beekeeping, poultry farming, and aquaculture [28]. The nearby areas include Chitwan National Park, Barandaabhar forest, community forests, and wetlands, supporting animals like one-horned rhinos, Asiatic elephants, Bengal tiger, deer, and avian species. Similarly, vegetation is dominated by Sal forest, riverine forest, mixed forest, and grasslands.

### 2.2. Sampling Population

**Figure 2:**
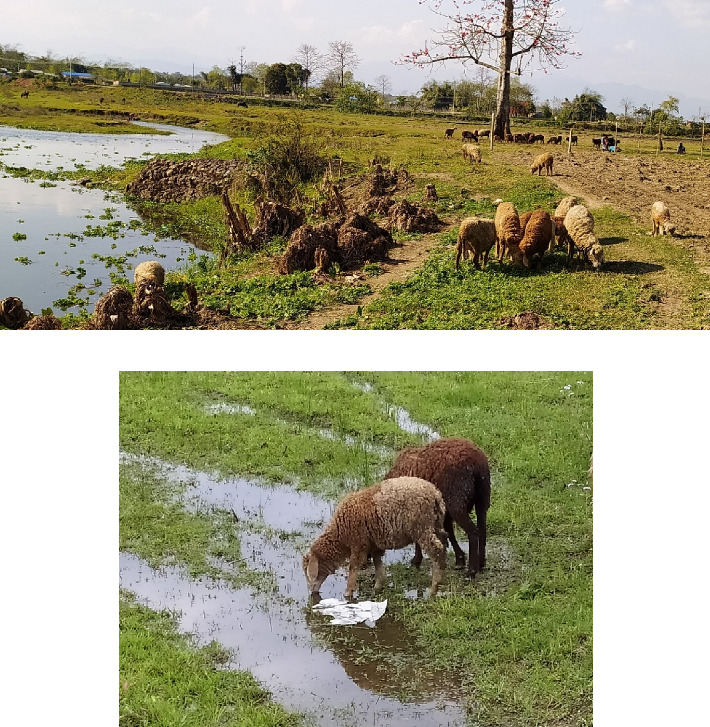
Sheep in the study area. (a) Sheep grazing nearby rivers and sharing grazing lands with buffaloes and goats. (b) Sheep drinking polluted water in wetlands.

This study was conducted within the agricultural regions in Ratnanagar. The sampling population of sheep belongs to the indigenous Kage breed (Figure 2), reared by ethnic communities like the Tharu, Kumal, and Magar people in Ratnanagar, Chitwan. Based on the Livestock Statistics of Nepal 2020/21, 3070 heads of sheep are found in Chitwan [3]. However, sheep farming in Ratnanagar is drastically reducing, and the estimated population is below 300. However, after visiting the 15 households in the core region, we collected fecal samples from 160 sheep (*N* = 160). Based on the literature [29], we categorized them into three age groups: lambs (less than 1 year; < 1 year), hoggets (equal or greater than 1 year but less than 2 years; ≥ 1-< 2 years), and adults (2 years or more, up to 10 years; ≥ 2–10 years). Among them, 73 individuals were male, and 87 individuals were female.

### 2.3. Fecal Sample Collection, Preservation, and Transportation

All fecal samples were collected using noninvasive techniques. Each sheep was observed in the pasture or farm until it defecated. Only the uncontaminated portion of the fallen fecal material was collected using a metallic spatula and gloved hands. We obtained verbal consent from sheep owners to note sheep's characteristics (age, sex, medication history, rearing practices, and others). To avoid the duplication of samples, particularly in the case of lambs, we provided red nail polish marks on sheep's legs. Before collecting samples into 20 mL sterile vials containing 2.5% weight/volume (w/v) potassium dichromate solution K_2_Cr_2_O_7_ (Sisco Research Laboratories Pvt. Ltd., India), they were inspected for fecal consistency, mucus, blood, and detached proglottids of tapeworms, and necessary photographs were taken. Then, the vials were transported to the Animal Research Laboratory, Nepal Academy of Science and Technology (NAST) for processing and microscopic analysis.

### 2.4. Laboratory Processing and Examination

We used four distinct methods to analyze fecal samples based on the previous protocol explained in the literature [30–34]. The fecal pellets were crushed using a glass rod and stirred vigorously in 2.5% K_2_Cr_2_O_7,_ which was used as the preservative medium.

#### 2.4.1. Direct Wet Mount

A single sample drop with or without Gram's iodine stain was placed on the glass slide. The smear was then covered with a coverslip and examined under a microscope (40x) (Optika Microscopes Italy, B-383PLi).

#### 2.4.2. Saturated Salt Flotation Technique

Initially, 13 mL of normal saline and 2 g of the fecal sample were transferred into a conical centrifuge tube and centrifuged for 5 minutes at 1200 revolutions per minute (rpm). The supernatant was disposed of, and the tube was centrifuged again (1200 rpm × 5 min) with flotation media (32% w/v ZnSO_4_). Then, without discarding the supernatant, additional flotation media was added drop by drop to fill the tube. Finally, a coverslip was placed at the tube's opening so that it touched the flotation media. After 10 minutes, the coverslip was gently removed and put on a glass slide for microscopic examination (40x).

#### 2.4.3. Formalin–Ethyl Acetate (FEA) Sedimentation

For this approach, 10% formalin (10 mL) and ethyl acetate (4 mL) were mixed with the sediment collected from a single centrifuge session. After that, the sample was centrifuged at 1200 rpm for 5 min, and the supernatant was discarded. Lastly, a sediment drop was examined under a microscope (40x).

#### 2.4.4. Acid-Fast Staining

For this technique, thin fecal smears were made using the sediment obtained after FEA sedimentation. Initially, the smear was dried at room temperature, fixed for 2 min in pure methanol, and then stained with carbol fuchsin for 15 min. After that, it was subsequently cleaned with distilled water and acid alcohol. Lastly, the smear was re-stained with malachite green for a minute and washed with distilled water. After complete air drying, it was examined under a microscope (100x) using immersion oil.

#### 2.4.5. Sporulation Assays

For this assay, each gram of *Eimeria* positive samples was mixed with 4 mL of 2.5% K_2_Cr_2_O_7_ solution in a Petri dish and incubated at 22–28°C for about a week with periodic examination of the sporulation state at each 24-h interval based on protocol [35, 36].

#### 2.4.6. Estimation of Parasitic Burden/Severity of Infection

We used an “A2 Cell McMaster Counting Slide” (Hawksley and Sons Ltd.) to calculate the burden of parasite infection by counting the number of oocysts and eggs released in every gram of feces. The process follows the guidelines the manufacturer's company instructed and the previously explained literature [37, 38].

### 2.5. Parasite Identification

All parasite stages, including cysts, oocysts, and trophozoites of protozoa, as well as eggs and larvae of helminths, were imaged under a compound microscope using a camera (SXView 2.2.0.172 Beta (Nov 6, 2014) Copyright (C) 2013-2014) attached to the microscope, and ImageJ 1.51 k (National Institute of Health, USA) software was used for morphometric analysis. All the parasitic stages, including the oocysts of *Eimeria* spp., were identified morphologically based on the previously published literature [12, 39–42]. The oocyst formula for *Eimeria* spp. is O.4.2, indicating that each sporulated oocyst contains four sporocysts, and each sporocyst houses two sporozoites [43]. Furthermore, *Fasciola* sp. and *Paramphistomum* sp. were detected using methylene blue staining. On staining with methylene blue, *Fasciola* spp. attain a golden yellow color, while *Paramphistomum* sp. remains colorless [44]. Furthermore, following the protocol of the literature [35], we did not utilize the fecal culture technique to identify nematode eggs up to third-stage larvae, a crucial step for a comprehensive diagnosis [45]. Therefore, Strongyle-type eggs of *Cooperia* sp., *Haemonchus* sp., *Oesophagostomum* sp., *Teladorsagia* sp., and *Trichostrongylus* sp., which all look pretty much the same, were considered as Strongyle.

### 2.6. Data Analysis

All the data generated during the study were encrypted in Microsoft Word 2016 and an Excel Sheet 2016. Prevalence rates of all reported parasites were calculated by dividing the number of positive cases (total or particular species) by the total number of samples collected and multiplying by 100. We used GraphPad Software (Prism 5 for Windows, Version 5.00@1992–2007, GraphPad Software, Inc.) to analyze the prevalence rates between different variables. For two variables, Fisher's exact test (two-sided) and the chi-square test were used to assess the *p*-value for more than two variables. Statistical significance was considered at a 95% confidence interval (*p* < 0.05). Furthermore, nonparametric correlation using the Spearman (r) (two-tailed) test was used to find the association concerning the pattern of parasitic infection between males and females among the three different age groups of sheep.

## 3. Results

### 3.1. Macroscopic Findings

**Figure 3:**
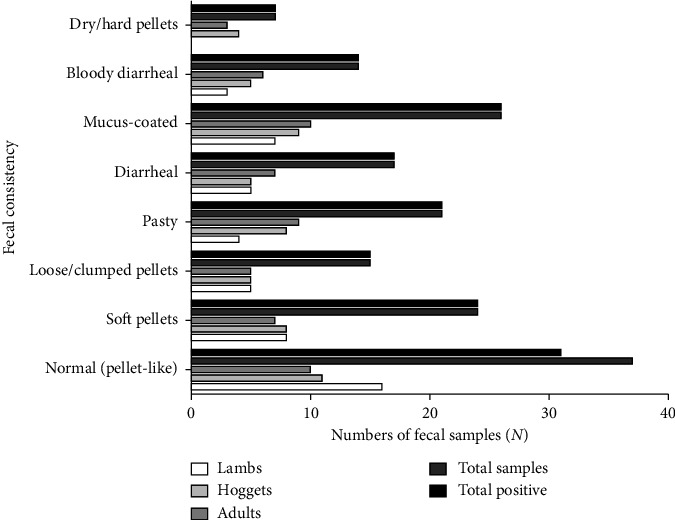
Fecal consistency and intestinal parasites.

The macroscopic examination revealed eight consistencies of fecal samples along different groups of sheep (Figure 3). It showed that maximum stool samples were of normal consistency (23.1%), followed by soft pellets (15%), and that of dry/hard pellets (4.4%) was the least (*p* < 0.05) (Figure 3) (Supporting Figure 1). Interestingly, all fecal samples, except for six-stool samples of normal consistency, were positive for parasites without any statistical significance (*p* > 0.05).

### 3.2. Microscopic Findings

**Figure 4:**
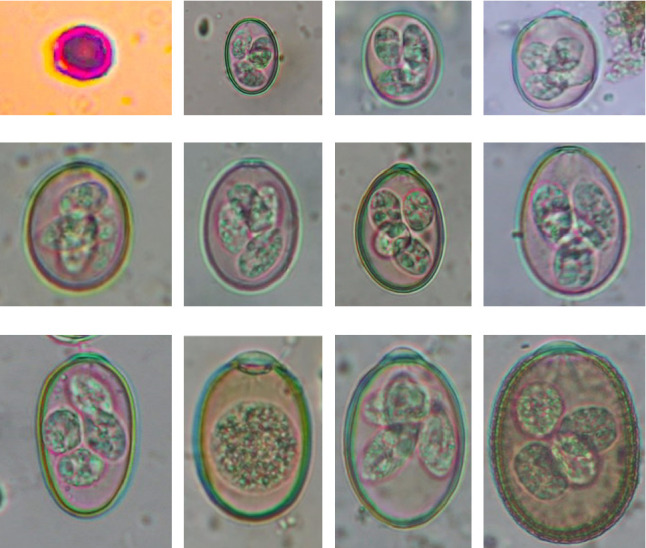
Oocysts of coccidian parasites. (a): *Cryptosporidium* sp., (6 × 6) μm. (b): *Eimeria pallida*, (21 × 15) μm. (c): *E. parva*, (24 × 18) μm. (d): *E. marsica*, (26 × 20) μm. (e): *E. ovinoidalis*, (27 × 20) μm. (f): *E. webridgensis*, (27 × 20) μm. (g): *E. crandallis*, (30 × 21) μm. (h): *E. faurei*, (32 × 22) μm. (i): *E. granulosa*, (36 × 21) μm. (j): *E. bakuensis*, (41 × 28) μm. (k): *E. ahsata*, (42 × 29) μm. (l): *E. intricata*, (54 × 36) μm.

In this study, the microscopic examination of fecal samples revealed that 154 out of 160 (96.3%) sheep were infected with at least a single species of intestinal parasites. Considering intestinal parasite diversity, sheep were more infected with protozoa (94.4%; 16 species) than helminth parasites (78.1%; 11 species). Protozoa included many coccidian parasites, such as *Cryptosporidium* sp. and 11 eimerian species. The prevalence of these species followed the order: *E. parva* (45.6%), *E. ahsata* (29.4%), *E. pallida* (28.1%), *E. faurei* (20%), *E. bakuensis* (20%), *E. webridgensis* (18.8%), *E. ovinoidalis* (17.5%), *E. marsica* (15%), *E. crandallis* (10.6%), *E. granulosa* (10.6%), and *E. intricata* (7.5%) (Figure 4) (Supporting Table 1). Age-wise prevalence was tested using chi-square tests, and significance was achieved only for *E. ahsata, E. faurei, E. marsica,* and *E. crandallis* (*p* < 0.05) (Table 1). Similarly, other protozoa included *Entamoeba* sp., *Blastocystis* sp., *Balantidium coli,* and *Giardia* sp. The prevalence rates of the former two were statistically different in different age groups of sheep (*p* < 0.05). Similarly, on comparing the rates of coccidia (84.4%) and other protozoa (65.6%), statistical significance (*p* < 0.05) was observed (Table 1).

**Figure 5:**
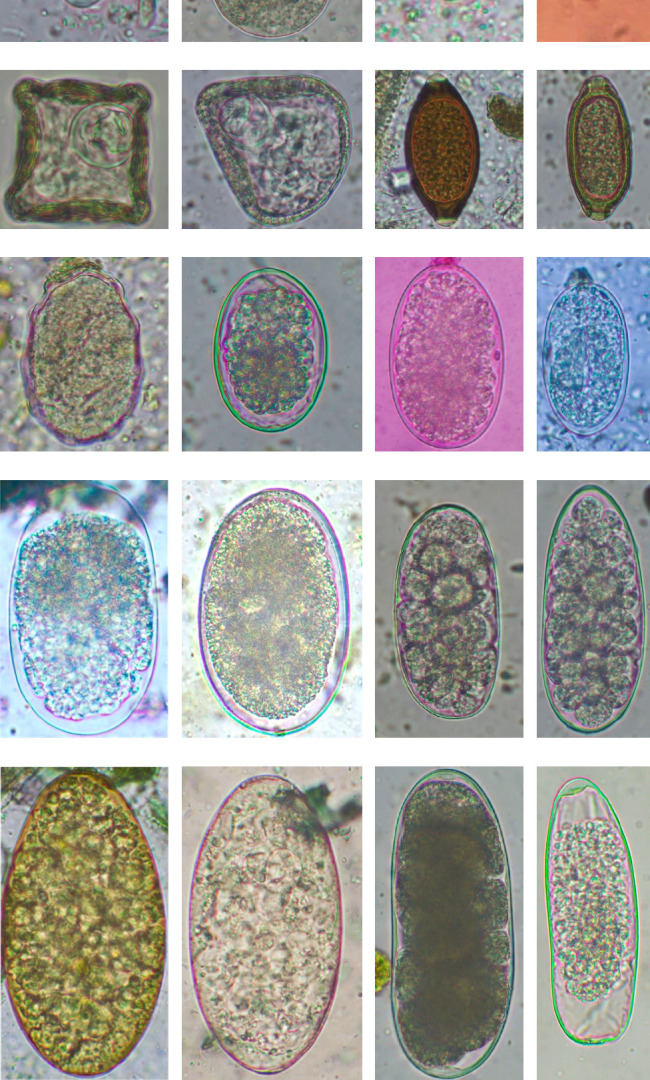
Morphological stages of intestinal protozoa and helminths in indigenous sheep. (a): Cyst of *Entamoeba* sp., (12 × 12) μm. (b): Cyst of *Balantidium coli*, (50 × 50) μm. (c): Cyst of *Blastocystis* sp., (12 × 12) μm. (d): Trophozoite of *Giardia* sp. (12 × 8) μm. (e): Egg of *Moniezia benedeni*, (48 × 46) μm. (f): Egg of *Moniezia expansa*, (68 × 65) μm. (g): Egg of *Trichuris ovis*, (64 × 31) μm. (h): Egg of *Capillaria* sp., (52 × 25) μm. (i): Egg of Ascarid sp., (66 × 42) μm. (j): Egg of Strongyle 1, (66 × 46) μm. (k): Egg of Strongyle 2, (76 × 47) μm. (l): Egg of *Strongyloides* sp., (66 × 42) μm. (m): Egg of Strongyle 3, (101 × 55) μm. (n): Egg of Strongyle 4, (104 × 67) μm. (o): Egg of Strongyle 5, (83 × 38) μm. (p): Egg of Strongyle 6, (108 × 42) μm. (q): Golden colored egg of *Fasciola* sp., (170 × 93) μm. (r): Colorless egg of *Paramphistomum* sp., (165 × 85) μm. (s): Egg of *Nematodirus* sp., (153 × 52) μm. (t): Egg of Strongyle 7, (114 × 39) μm.

Interestingly, total trematodes, as well as individual trematode parasites (*Fasciola* sp. and *Paramphistomum* sp.), revealed a significant difference in prevalence (*p* < 0.05). In contrast, two cestodes (*Moniezia expansa* and *M. benedeni*), observed eggs microscopically and proglottids macroscopically, were not different statistically (*p* > 0.05). Furthermore, we reported five morphotypes of the Strongyle-type eggs with sizes ranging (63–114 × 39–67 μm). Interestingly, Strongyles were the principal helminths present in all age groups of sheep, although the prevalence rates were insignificant (*p* > 0.05). The rates of overall helminths and *Nematodirus* sp. were different concerning sheep ages (*p* < 0.05), and other detected nematodes like Ascarid sp., Strongyle, *Strongyloides* sp., *Capillaria* sp., and *Trichuris ovis* were not statistically significant (*p* > 0.05) (Table 1) (Figure 5). Notably, the prevalence rates of cestodes and nematodes among different age groups of sheep were found to be statistically insignificant (*p* > 0.05).

The study analyzed the age-based differences in parasite prevalence, diversity, and load. Interestingly, helminth parasites, like Ascarid sp. and *Nematodirus* sp., were only found in the lambs, while protozoa, like *Balantidium* sp., were only reported from adult sheep. Similarly, adult sheep showed a higher prevalence rate and diversity (100%; 24 spp.) of intestinal parasites than other lambs (91.7%; 21 spp.) and hoggets (96.3%; 23 spp.). Regarding coccidian, each age group of the sheep harbored all 12 species of parasites with a higher prevalence rate in adults (93%) than hoggets (80%) and lambs (79.2%); however, the parasitic load was higher in lambs (OPG: 400–13,500) and hoggets (OPG: 300–12,500) than adults (OPG: 200–7200) as revealed by McMaster technique (Supporting Table 2). Additionally, all age groups of sheep released the highest number of oocysts of *Eimeria* spp. (OPG: 300–13,500) and Strongyle egg (EPG: 200–9500) in their feces (Supporting Table 2).

**Figure 6:**
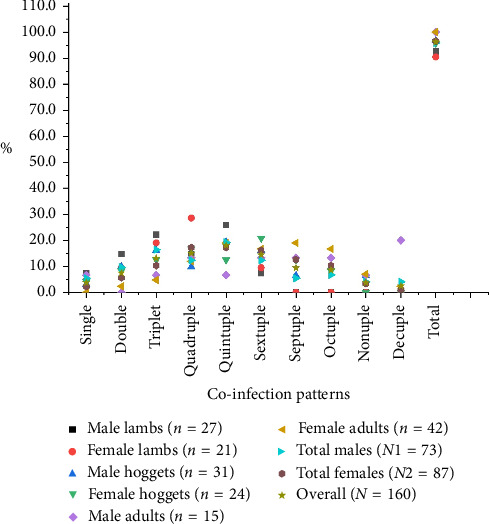
Co-infection of intestinal parasites in different ages and sexes of sheep.

Considering the concurrency of parasitic infections, polyparasitism (92.5%) was significantly higher than monoparasitism (3.8%) (*p* < 0.005), and concurrency with a maximum of 10 species of parasites at a time was recorded. The quintuple pattern of infection was highest in lambs, while hoggets and adults were co-infected with sextuple and septuple patterns of infection, respectively (Figure 6). Additionally, the Spearman correlation test (*r*) indicated a significant association in the pattern of parasitic coinfections between male and female lambs (*r* = 0.928, *p* < 0.05) and hoggets (*r* = 0.7201, *p* < 0.05), or total male and female sheep (*r* = 0.7662, *p* < 0.05); however, such an association was not observed among adults (*r* = 0.28, *p* > 0.05). Similarly, the chi-square test confirmed statistical significance (*p* < 0.05) in analyzing total positive samples at various concurrency levels. Based on sex, female sheep (96.6%) had a higher prevalence of intestinal parasites than males (95.9%). Notably, while parasites were present in all adult male and female sheep, they were more prevalent in male lambs (92.6% vs. 90.5%) and male hoggets (96.8% vs. 95.8%) than their female counterparts (Figure 6).

Prevalence data and statistical analyses by chi-square tests and Fisher's exact tests revealed that grazing practices, domestication practices, and medication history were the risk factors of GI parasitosis among the studied sheep (*p* < 0.05). However, sheep musculature, drinking water sources, and types of the resting floor were not associated with GI parasites (*p* > 0.05) (Table 2).

## 4. Discussion

This study was the first to examine diversity, prevalence rates, concomitance, and risk factors of intestinal parasites in sheep in the lowlands of Nepal. The prevalence rate of the GI parasites in the sheep (96.3%, 154/160) was slightly higher than findings reported in locally published papers in Nepal, ranging from 54% (*n* = 110) to 80% (*n* = 150) [11, 25]. Conversely, our prevalence rate was lower than those documented in several other countries: India (85.2%; *n* = 391) [46], China (55.7%; *n* = 246) [14], Bangladesh (77.1%; *n* = 572) [47], Pakistan (94%; *n* = 184) [48], Iran (53.3%; *n* = 2040) [15], Brazil (82.03%; *n* = 807) [49], and Lesotho (89.2%; *n* = 1804) [50], suggesting a global variability in GI parasite prevalence across different agricultural regions. In this scenario, several factors may rule out these differences. This may include factors such as the surrounding environment (climate, soil, and pasture conditions), grazing management practices (including interactions between wildlife and livestock), seasonal variations, the sampling breed and health of the animals, adopted deworming practices, nutritional status, and the overall management system. In our cases, sampling has been conducted from the indigenous breed of sheep, and statistical analysis has revealed a significant association of parasitism with grazing practices, domestication practices, and medication history.

In the current study, the prevalence and diversity of protozoan parasites were higher than helminths, and this was primarily due to coccidian parasites. Among these, *Eimeria* spp. were highly predominant across all age groups of sheep. In line with findings from Egypt [40] and Estonian Island [41], we reported 11 morphotypes of these coccidia (Figure 4), highlighting their predominance and extensive diversity in sheep from Nepal. Although *E. ovinoidalis* is considered to be the most pathogenic, other species, like *E. ahsata, E. crandallis, E. parva, E. marsica, E. bakuensis,* and *E. granulosa,* have also been reported to cause mild coccidiosis. These species cause substantial intestinal damage and clinical symptoms, like diarrhea, weight loss, dehydration, and occasionally death, particularly in young, growing, or stressed animals [12, 51–54], signifying a critical role of these coccidians in sheep health in Nepal.

Generally, veterinarians and parasitologists often use the term “coccidiosis” to refer to infections caused by *Eimeria* species exhibiting high host specificity. However, more caution is warranted due to the pathogenic and zoonotic potential of another coccidian of the genus *Cryptosporidium*. With strains like *Cryptosporidium parvum*, *C. ubiquitum*, and *C. xiaoi* being the most common, other species like *C. andersoni*, *C. bovis*, *C. scrofarum*, and *C. suis* have also been isolated from sheep from different geographies [55–57]. Therefore, in this circumstance, particularly in rural settings, many factors—such as close contact between humans and infected sheep, both indoors and outdoors, lack of proper farm sanitation, and hygienic farming practices, including the improper disposal of animal waste and the provision of water from contaminated sources, along with farmers' behavior of walking barefoot and not using gloves and boots while working on the farm—may increase the likelihood of zoonosis of these coccidia. Notably, the prevalence rate of these coccidia in the currently sampled sheep (23.1%) was slightly higher than those reported in Poland (19.3%) [55] and Australia (16.9%) [56]. *Cryptosporidium* spp. mainly affect young, elderly, and immunocompromised individuals, causing intestinal damage, malabsorption, diarrhea, and dehydration, resulting in death at extreme infection [13, 58]. However, the impact of cryptosporidiosis caused by these coccidia on the Nepalese sheep population has not yet been thoroughly investigated, particularly due to the lack of routine examinations of the oocysts in veterinary and medical laboratories. Therefore, further molecular and histopathological research is needed for species identification, evaluation of clinical pathogenesis, and zoonotic potential of various *Cryptosporidium* species.

As Strongyles were the most predominant helminths (74%) in the studied sheep, a similarly high prevalence of Strongyles, ranging from 59.2% to 84.1%, has also been reported in India [59]. GI infection with Strongyle worms, like *Telodorsagia*, *Trichostrongylus* spp., and *Haemonchus contortus*, mainly causes serious health issues in sheep. The former two cause gastroenteritis in growing lambs and contribute to immunosuppression during concurrent infections in older animals [60]. At the same time, *Haemonchus contortus* can lead to severe anemia, lethargy, emaciation, bottle jaw, and, in severe cases, collapse and death [60, 61].

On the other hand, although *Cooperia* and *Oesophagostomum* are not as lethal or aggressive as mentioned earlier, they can still compromise health and increase the pathogenic burden, particularly in younger or malnourished animals during multiparasitism [62]. We detected three lambs infected with *Nematodirus* sp. The nematode primarily affects lambs and may lead to weight loss, severe diarrhea, and significant dehydration [62]. Alarmingly, nematodirosis has been linked to nearly 20% mortality in infected flocks [62], highlighting its profound impact on the sheep population. The strongylid predominance in the current study might have occurred due to grazing access provided on wetland and crop residue areas. Additionally, the warmer and more humid conditions in the lowland area also promote the development and survival of their larvae [59].

Considering cestodes, both *Moniezia expansa* and *M. benedeni* were present in the fecal samples. These tapeworms rarely cause clinical or economic burdens to adults. However, they may impart pathogenic symptoms, like potbellies, constipation or mild diarrhea, poor growth, rough coat, and anemia in young animals [63]. Since the current population of sheep graze on the wetlands and engage in stubble grazing, they might have acquired the infection via the ingestion of soil mites (*Galumna* spp. and *Oribatula* spp.), which thrive in moist environments and serve as intermediate hosts [63, 64].

The study indicated that trematodes like *Paramphistomum* sp. and *Fasciola* sp. are important in ovine populations. Interestingly, both parasites pose significant risks to sheep, primarily affecting the liver and GI system, resulting in reduced productivity and death [65]. Since the field survey detected freshwater snails, including Planorbid and Lymnaeid species, the intermediate hosts of those flukes, in the nearby water bodies and wetlands [66], the grazing sheep ingest the infective metacercaria larva, resulting in the acquisition of higher infection rates.

Furthermore, in line with the findings from China [14], we reported a higher prevalence of parasite infection in females than in males. This may be due to the unbalanced gender sample size and the large proportion of adult females sampled in the current study. It is speculated that the physiological changes during pregnancy and breastfeeding, combined with the physical and metabolic demands of pregnancy and raising lambs, make females more susceptible to parasitic infections [67, 68]. As a result, helminth-infested animals, accompanied by reduced immunity, may increase egg production. In addition, pasturelands are the best sources of overwintered helminth larvae; for example, Strongyle larvae have the potential to overwinter and infect the available hosts [69].

Similarly, based on age, we observed that adult sheep have a higher prevalence rate of parasites than lambs and hoggets. However, our findings contradicted the findings from Bangladesh [47], which reported a higher prevalence rate in lambs. Even though a few studies claimed that the immature immune systems in lambs make them susceptible to parasites [47, 70], others explained a consistent composition of parasites in both adults and lambs, suggesting both can experience higher infection rates during humid and rainy seasons [50]. In this context, it is believed that the traditional rearing practices, repeated exposure to parasites and contaminated pastures and wetlands, might have added infection risk to adults. In contrast, newly born lambs were provided intense care and remained separated from the adult flock in a pen or resting place.

Considering various risk factors, providing drinking water from contaminated sources or allowing sheep to access outdoor water bodies can be a significant risk. As outdoor water sources usually remain contaminated with the feces of the sheep or other animals, they serve as reservoirs for waterborne parasites such as *Giardia* sp., *Entamoeba* spp., *Cryptosporidium* sp., *Fasciola* spp., *Paramphistomum* sp., and *Schistosoma* spp. [71, 72]. In these situations, sheep can ingest the infectious stages of these parasites via drinking contaminated water or grazing in infected pastureland; interestingly, these sources can be vital for the parasite reinfection cycle.

Similarly, we observed that sheep populations with the absence of deworming practices or irregular deworming had higher prevalence rates of parasites. Our communication with sheep owners also revealed that they follow medication or deworming based on information provided by herders rather than relying on scientific laboratory diagnoses of the parasites. In this scenario, dosing errors and incomplete courses lead to reduced efficacy, suggesting that a lack of effective deworming might be a significant risk for parasitosis in the study area. Furthermore, inconsistent deworming practices lead to increased reinfection rates, implying individuals with higher initial infection burdens are more susceptible to reinfection in the absence of regular deworming [73]. In addition, such practices also allow incomplete parasite elimination and may create a suitable environment for the parasites to accumulate in the animals and pastures [74, 75]. It also hinders the development of natural immunity in lambs and leaves the flock unprotected during high-risk periods, especially in the warm and wet seasons [76, 77]. Importantly, in Nepal, veterinarians usually do not follow the diagnosis methods of cryptosporidiosis, and there is no question about treating this infection in the sheep population, which enhances their prevalence rates among these host populations.

We also reported that polyparasitism (92.5%) is predominantly present across all age groups of sheep, with a maximum concurrency of up to 10 species at a time. This finding is consistent with research from China [14], which also noted similar infection patterns. In general, co-infections with multiple parasite species, including other pathogens within the same host, are common, and this interaction may result in positive, negative, or neutral effects on the infections [78]. For instance, our previous study reported a robust pathology in a buffalo calf heavily infected with multiple species of protozoa and helminth parasites [79]. Additionally, co-infection of Yersiniosis (Bacteria) and nematodiasis (internal parasitism) in lambs has been linked to increased mortality rates in sheep [80, 81], implying that polyparasitism enhances the host's susceptibility to secondary infection caused by bacteria and viruses [82].

When considering co-infections with protozoa and helminths in sheep, there is evidence of significant synergistic effects of coccidiosis and strongylosis, potentially resulting in a higher mortality rate [83]. This remains true for individuals with immunocompromised states, those exposed to harsh environmental conditions, or those suffering from malnutrition [22, 84]. This evidence suggests that the current sheep population that is heavily co-infected with pathogenic *Eimeria* spp. and Strongyle worms, 41% in lambs, 56.4% in hoggets, and 61.4% in adults and excreting moderate to high levels of OPG/EPG of these parasites, as suggested by McMaster count, might have a substantial impact on their GI health. However, a histopathological finding in highly infected individuals will be crucial for understanding the clinical effects of co-infections in sheep in Nepal.

## 5. Conclusions

In conclusion, the current study has uncovered the diversity, loads, and prevalence of GI parasitism in indigenous sheep on smallholder farms in central Nepal. Our findings indicate that parasitism is associated with individual characteristics such as age and sex, as well as environmental and management factors, including grazing access, mixed domestication practices, and deworming practices. Since these GI parasites can ultimately lead to illnesses and impact the survival of sheep, particularly in the absence of high care, medications, and prompt health management, early diagnosis of the parasites, including that of pathogenic species, is crucial. Although *Cryptosporidium* is often overlooked in coccidiosis, it should be routinely included alongside major coccidian species, such as the Eimerian parasites, to enhance the welfare, productivity, and sustainability of sheep farming in Nepal. Therefore, the concerned authorities and government organizations, including state and private veterinarians, must prioritize multilevel strategies that involve community education, hygiene promotion, and veterinary care to mitigate the risk effectively. Therefore, routine screening for early detection of coccidian and other GI parasites and the rational, targeted use of antiparasitic drugs must be ensured for the successful management of sheep farms in the country.

## Figures and Tables

**Table 1:** Age-wise prevalence of intestinal parasites in indigenous sheep (*N* = 160).

Class	Parasites	Lambs (< 1 year) *n*1 = 48	Hoggets (≥ 1–< 2 years) *n*2 = 55	Adults (≥ 2–10 years), *n*3 = 57	Overall (*N* = 160)	*p*-values (age-wise)
*Protozoa*						
Sarcodina	*Entamoeba* sp.	23 (47.9)	38 (69.1)	41 (71.9)	102 (63.8)	*p* < 0.05
	*Blastocystis* sp.	1 (2.1)	1 (1.8)	1 (1.8)	3 (1.9)	*p* < 0.05
Mastigophora	*Giardia* sp.	4 (8.3)	4 (7.3)	6 (10.5)	14 (8.8)	ns
Ciliata	*Balantidium coli*	0 (0)	0 (0)	1 (1.8)	1 (0.6)	ns
Sporozoa	*Cryptosporidium* sp.	10 (20.8)	11 (20)	16 (28.1)	37 (23.1)	ns
	*Eimeria pallida*	12 (25)	15 (27.3)	18 (31.6)	45 (28.1)	ns
	*E. parva*	17 (35.4)	26 (47.3)	30 (52.6)	73 (45.6)	ns
	*E. marsica*	2 (4.2)	8 (14.5)	14 (24.6)	24 (15)	*p* < 0.05
	*E. ovinoidalis*	5 (10.4)	8 (14.5)	15 (26.3)	28 (17.5)	ns
	*E. webridgensis*	8 (16.7)	7 (12.7)	15 (26.3)	30 (18.8)	ns
	*E. crandallis*	1 (2.1)	6 (10.9)	10 (17.5)	17 (10.6)	*p* < 0.05
	*E. faurei*	6 (12.5)	8 (14.5)	18 (31.6)	32 (20)	*p* < 0.05
	*E. granulosa*	2 (4.2)	6 (10.9)	9 (15.8)	17 (10.6)	ns
	*E. bakuensis*	9 (18.8)	7 (12.7)	16 (28.1)	32 (20)	ns
	*E. ahsata*	9 (18.8)	15 (27.3)	23 (40.4)	47 (29.4)	*p* < 0.05
	*E. intricata*	4 (8.3)	4 (7.3)	4 (7)	12 (7.5)	ns
Total	Coccidian	38 (79.2)	44 (80)	53 (93)	135 (84.4)	ns
	Other protozoa	25 (52.1)	38 (69.1)	42 (73.7)	105 (65.6)	ns

*Helminths*						
Trematoda	*Fasciola* sp.	0 (0)	9 (16.4)	20 (35.1)	29 (18.1)	*p* < 0.05
	*Paramphistomum* sp.	0 (0)	22 (40)	29 (50.9)	51 (31.9)	*p* < 0.05
Cestoda	*Moniezia benedeni*	2 (4.2)	5 (9.1)	2 (3.5)	9 (5.6)	ns
	*M. expansa*	0 (0)	3 (5.5)	3 (5.3)	6 (3.8)	ns
Nematoda	Ascarid sp.	2 (4.2)	0 (0)	0 (0)	2 (1.3)	ns
	Strongyle except *Nematodirus*	30 (62.5)	42 (76.4)	46 (80.7)	118 (73.8)	ns
	*Strongyloides* sp.	12 (25)	12 (21.8)	15 (26.3)	39 (24.4)	ns
	*Nematodirus* sp.	3 (6.3)	0 (0)	0 (0)	3 (1.9)	*p* < 0.05
	*Capillaria* sp.	0 (0)	1 (1.8)	2 (3.5)	3 (1.9)	ns
	*Trichuris ovis*	2 (4.2)	4 (7.3)	3 (5.3)	9 (5.6)	ns
Total	Trematoda	0 (0)	24 (43.6)	34 (59.6)	58 (36.3)	*p* < 0.05
	Cestoda	2 (4.2)	6 (10.9)	5 (8.8)	13 (8.1)	ns
	Nematoda	33 (68.6)	44 (80)	50 (87.7)	127 (79.4)	ns
Grand total	Overall protozoa	44 (91.7)	52 (91.2)	55 (96.5)	151 (94.4)	ns
	Overall helminths	33 (68.8)	47 (85.5)	53 (93)	133 (83.1)	*p* < 0.05
	Overall parasites	44 (91.7)	53 (96.3)	57 (100)	154 (96.3)	ns

**Table 2:** Assessment of risk factors/predictors of risk **(**Fisher's exact test for two variables and the chi-square test for more than two variables have been used).

Risk factors	Predictor of risks	No. of sheep	Prevalence (%)	Statistics (*p*-values)
Grazing practices	Grazers	132	131 (99.2)	*p* < 0.05
	Non-grazers	28	23 (82.1)	

Musculature	Normal	122	116 (95.1)	ns
	Thin and skinny	31	31 (100)	
	Weak	7	7 (100)	

Drinking water	Indoor	74	70 (94.6)	ns
	Outdoor access (water bodies)	86	84 (97.7)	

Domestication practices	Single	6	4 (66.7)	*p* < 0.05
	Multiple	98	94 (95.9)	
	Mixed with other livestock	56	56 (100)	

Pen (floor)	Cemented	12	11 (91.7)	ns
	Mud	32	32 (100)	
	Wooden	116	111 (95.7)	

Medication history	≤ 6 months	26	22 (84.6)	*p* < 0.05
	7–12 months	38	36 (94.7)	
	> 12 months/unknown history	96	96 (100)	

## Data Availability

The data that support the findings of this study are available upon request from the corresponding author. The data are not publicly available due to privacy or ethical restrictions.

## Supporting Information

Supporting Figure 1: Fecal consistency of indigenous sheep.

Supporting Table 1: Oocyst characteristics of *Eimeria* spp. in indigenous sheep (*n* = number of oocyst/sporozoite measured).

Supporting Table 2: OPG/EPG of coccidian and nematode parasites in the fecal samples concerning the age of the sheep.

## References

[1] Kochewad S. A., Meena L. R., Kumar S., Kumar V., Meena L. K. (2017). Sheep Rearing Systems and Their Productive Performances–A Review. *Trends in Biosciences*.

[2] Dahal B. P. (2020). Dialectics of Sacrificing and Worshiping Animals in Hindu Festivals of Nepal. *Advances in Anthropology*.

[3] MoALD (2023). *Statistical Information on Nepalese Agriculture 2078/79 (2021/22)*.

[4] Gorkhali N. A., Sapkota S., Bhattarai N., Pokhrel B. R., Bhandari S. (2021). *Indigenous Livestock Breeds of Nepal: A Reference Book*.

[5] Shrestha B. S., Shrestha S., Neopane S. P., Shrestha N. P. Sheep Genetic Resources of Nepal.

[6] Rauniyar G. P., Upreti C. R., Gavigan R., Parker W. J. (2000). Constraints to Sheep Farming in Nepal: Development Challenge for Poverty Alleviation. *Asian-Australasian Journal of Animal Sciences*.

[7] Rss (2023). Sheep Farming Facing Threat as New Generation Not Showing Interest. *Rastriya Samachar Samiti*.

[8] Ghimire T. R., Regmi G. R., Huettmann F., Regmi G. R., Huettmann F. (2020). When Micro Drives the Macro: A Fresh Look at Disease and Its Massive Contributions in the Hindu Kush-Himalaya. *Hindu Kush-Himalaya Watersheds Downhill: Landscape Ecology and Conservation Perspectives*.

[9] Sapkota A., Sapkota B., Khatiwada S., Neupane G. (2020). Transhumant Goat and Sheep Husbandry Practices in High Hills of Annapurna Conservation Area. *IJEAB*.

[10] Rokaya J. B. (2016). Viral Disease Affecting Goats and Sheep Spreads to Humla. *The Kathmandu Post*.

[11] Shrestha A., Karmacharya R. (2016). Epidemiological Study of Diseases Outbreak in Sheep of Lumle, Nepal. *International Journal of Veterinary Sciences and Animal Husbandry*.

[12] Chartier C., Paraud C. (2012). Coccidiosis Due to *Eimeria* in Sheep and Goats, A Review. *Small Ruminant Research*.

[13] Foreyt W. J. (1990). Coccidiosis and Cryptosporidiosis in Sheep and Goats. *Veterinary Clinics of North America: Food Animal Practice*.

[14] Cai W., Cheng C., Feng Q. (2023). Prevalence and Risk Factors Associated with Gastrointestinal Parasites in Goats (*Capra hircus*) and Sheep (*Ovis aries*) from Three Provinces of China. *Frontiers in Microbiology*.

[15] Salehi A., Razavi M., Vahedi Nouri N. (2022). Seasonal Prevalence of Helminthic Infections in the Gastrointestinal Tract of Sheep in Mazandaran Province, Northern Iran. *Journal of Parasitology Research*.

[16] Halvarsson P., Höglund J. (2021). Sheep Nemabiome Diversity and Its Response to Anthelmintic Treatment in Swedish Sheep Herds. *Parasites & Vectors*.

[17] Scholthof K. B. G. (2007). The Disease Triangle: Pathogens, The Environment and Society. *Nature Reviews Microbiology*.

[18] Jacobson C., Larsen J. W., Besier R. B., Lloyd J. B., Kahn L. P. (2020). Diarrhoea Associated With Gastrointestinal Parasites in Grazing Sheep. *Veterinary Parasitology*.

[19] Ramabu S. S., Tlotleng K., Mosweu T. M. (2018). After Tropical Cyclone Dineo an Increased Mortality in Sheep and Goats at Botswana University of Agriculture and Natural Resources (Buan) Farm. *International Journal of Food, Agriculture and Veterinary Sciences I*.

[20] Roeber F., Jex A. R., Gasser R. B. (2013). Impact of Gastrointestinal Parasitic Nematodes of Sheep, and the Role of Advanced Molecular Tools for Exploring Epidemiology and Drug Resistance-An Australian Perspective. *Parasites & Vectors*.

[21] Hadgu A., Lemma A., Yilma T., Fesseha H. (2021). Major Causes of Calf and Lamb Mortality and Morbidity and Associated Risk Factors in the Mixed Crop-Livestock Production System in Jamma District, South Wollo, Ethiopia. *Veterinary Medicine International*.

[22] Mlimbe M. E., Hyera E., Ochanga P. O. (2020). Study on the Causes and Pattern of Sheep Mortality Under Farm Conditions in Northern Tanzania. *Livestock Research for Rural Development*.

[23] Gangane G. R., Narladkar B. W., Moregaonkar S. D. (2019). Concurrent Endo and Ecto Parasitic Infections in Deccani Sheep. *Journal of Entomology and Zoology Studies*.

[24] Amaravathi M., Reddy B. K., Reddy N. (2016). A Case Report of Amphistomiasis in Sheep. *International Journal of Advanced Multidisciplinary Research*.

[25] Rana G., Subedi J. R. (2023). Gastro-intestinal Parasites of Sheep (*Ovis aries*, Linnaeus, 1758) in Laxmipur VDC, Dang, Nepal. *Nepalese Veterinary Journal*.

[26] Hananeh W. (2020). Postmortem Evidence of Abomasal Coccidiosis in Awassi Lamb in Jordan. *Veterinary Medicine and Public Health Journal*.

[27] Craig B. H., Pilkington J. G., Pemberton J. M. (2006). Gastrointestinal Nematode Species Burdens and Host Mortality in a Feral Sheep Population. *Parasitology*.

[28] Adhikari R. B., Dhakal M. A., Ale P. B., Regmi G. R., Ghimire T. R. (2023). Survey on the Prevalence of Intestinal Parasites in Domestic Cats (*Felis catus* Linnaeus, 1758) in Central Nepal. *Veterinary Medicine and Science*.

[29] Ponnampalam E. N., Holman B. W. B., Scollan N. D. (2016). Sheep: Meat. *Encyclopedia of Food and Health*.

[30] Sapkota B., Adhikari R. B., Regmi G. R., Bhattarai B. P., Ghimire T. R. (2020). Diversity and Prevalence of Gut Parasites in Urban Macaques. *Applied Science and Technology Annals*.

[31] Adhikari R. B., Parajuli R. P., Maharjan M., Ghimire T. R. (2021). Prevalence and Risk Factors of Gastrointestinal Parasites in the Chepangs in Nepal. *Annals of parasitology*.

[32] Adhikari R. B., Ale P. B., Dhakal M. A., Ghimire T. R. (2022). Prevalence and Diversity of Intestinal Parasites in Household and Temple Pigeons (*Columba livia*) in Central Nepal. *Veterinary Medicine and Science*.

[33] Aryal M., Adhikari R. B., Kandel P. (2022). First Report on the Molecular Detection of *Entamoeba Bovis* From the Endangered Wild Water Buffalo (*Bubalus arnee*) in Nepal. *Veterinary Medicine and Science*.

[34] Adhikari R. B., Ghimire D., Ghimire T. R. (2025). Investigation of the Occurrence of Zoonotic Intestinal Parasites along the Karmanasa River Bank in Lalitpur, Nepal, Nepal. *Veterinary Medicine and Science*.

[35] Adhikari R. B., Maharjan M., Ghimire T. R. (2020). Prevalence of Gastrointestinal Parasites in the Frugivorous and the Insectivorous Bats in Southcentral Nepal. *Journal of Parasitology Research*.

[36] Ghimire T. R., Adhikari R. B., Bhattarai N. (2022). Diversity and Prevalence of *Eimeria* Species in Goats of Nepal. *Journal of the Hellenic Veterinary Medical Society*.

[37] Adhikari R. B., Dhakal M. A., Ghimire T. R. (2023). Prevalence of Intestinal Parasites in Street Dogs (*Canis lupus Familiaris*) With Highlights on Zoonosis in Lalitpur, Nepal. *Veterinary Medicine and Science*.

[38] Adhikari R. B., Adhikari Dhakal M., Thapa S., Ghimire T. R. (2021). Gastrointestinal Parasites of Indigenous Pigs (*Sus domesticus*) in South‐Central Nepal. *Veterinary Medicine and Science*.

[39] Soulsby E. J. (2012). *Helminths, Arthropods and Protozoa of Domestic Animals*.

[40] El-Alfy E. S., Abbas I., Al-Kappany Y., Al-Araby M., Abu-Elwafa S., Dubey J. P. (2020). Prevalence of *Eimeria* Species in Sheep (*Ovis aries*) From Dakahlia Governorate, Egypt. *Journal of Parasitic Diseases*.

[41] Lassen B., Järvis T., Mägi E. (2013). Gastrointestinal Parasites of Sheep on Estonian Islands. *Agraarteadus: Journal of Agricultural Science*.

[42] Zajac A., Conboy G. A. (2012). *Veterinary Clinical Parasitology*.

[43] Ghimire T. R. (2010). Redescription of Genera of Family Eimeriidae Minchin, 1903. *International Journal of Life Sciences*.

[44] Adhikari R. B., Adhikari Dhakal M., Ghimire T. R. (2022). Prevalence and Diversity of Gastrointestinal Parasites in Domestic Buffaloes (*Bubalus bubalis* Linnaeus, 1758) Reared Under Captive and Semi-Captive Conditions in Ratnanagar, Chitwan, Nepal. *Annals of Parasitology*.

[45] Sabatini G. A., De Almeida Borges F., Claerebout E. (2023). Practical Guide to the Diagnostics of Ruminant Gastrointestinal Nematodes, Liver Fluke and Lungworm Infection: Interpretation and Usability of Results. *Parasites & Vectors*.

[46] Singh E., Kaur P., Singla L. D., Bal M. S. (2017). Prevalence of Gastrointestinal Parasitism in Small Ruminants in Western Zone of Punjab, India. *Veterinary World*.

[47] Dey A. R., Begum N., Biswas H., Alam M. Z. (2021). Prevalence and Factors Influencing Gastrointestinal Parasitic Infections in Sheep in Bangladesh. *Annals of parasitology*.

[48] Ruhoollah K. W., Khan W., Al-Jabr O. A. (2023). Prevalence of Gastrointestinal Parasite in Small Ruminants of District Dir Upper Khyber Pakhtunkhwa Province of Pakistan. *Brazilian Journal of Biology*.

[49] Carneiro P. G., Sasse J. P., Silva A. C. D. S. (2022). Prevalence and Risk Factors of *Eimeri*a Spp. Natural Infection in Sheep From Northern Paraná, Brazil. *Revista Brasileira de Parasitologia Veterinaria*.

[50] Phalatsi M. S., Seloanyane M., Motente M. (2022). Prevalence and Seasonal Abundance of Gastrointestinal Parasites of Merino Sheep in Maseru District, Lesotho. *Animal and Veterinary Sciences*.

[51] Andrews A. (2022). *Coccidiosis of Sheep*.

[52] Al-Neama R. T., Bown K. J., Blake D. P., Birtles R. J. (2021). Determinants of *Eimeria* and *Campylobacter* Infection Dynamics in UK Domestic Sheep: The Role of Co-Infection. *Parasitology*.

[53] Skirnisson K. (2007). *Eimeria* spp. (Coccidia, Protozoa) Infections in a Flock of Sheep in Iceland: Species Composition and Seasonal Abundance. *Icelandic Agricultural Sciences*.

[54] Gregory M. W., Catchpole J. (1990). Ovine Coccidiosis: The Pathology of *Eimeria crandallis* Infection. *International Journal for Parasitology*.

[55] Kaupke A., Michalski M. M., Rzeżutka A. (2017). Diversity of *Cryptosporidium* Species Occurring in Sheep and Goat Breeds Reared in Poland. *Parasitology Research*.

[56] Yang R., Jacobson C., Gardner G., Carmichael I., Campbell A. J. D., Ryan U. (2014). Longitudinal Prevalence, Oocyst Shedding and Molecular Characterisation of *Eimeria* Species in Sheep across Four States in Australia. *Experimental Parasitology*.

[57] Chen Y., Qin H., Huang J., Li J., Zhang L. (2022). The Global Prevalence of *Cryptosporidium* in Sheep: A Systematic Review and Meta-Analysis. *Parasitology*.

[58] Dahmani H., Ouchene N., Dahmani A., Ouchene-Khelifi N. A., Oumouna M. (2020). First Report on *Cryptosporidium parvum, Escherichia coli* K99, Rotavirus and Coronavirus in Neonatal Lambs From North-Center Region, Algeria. *Comparative Immunology, Microbiology and Infectious Diseases*.

[59] Swarnkar C. P., Singh D. (2014). Influence of Annual Rainfall on Epidemiology of Gastrointestinal Parasites in Sheep Flocks of Rajasthan. *Indian Journal of Animal Sciences*.

[60] McCarter P. (2019). *Gastrointestinal Nematode Infestations in Sheep*.

[61] Yacob H. T., Mistre C., Adem A. H., Basu A. K. (2009). Parasitological and Clinical Responses of Lambs Experimentally Infected With *Haemonchus contortus* (L3) With and Without Ivermectin Treatment. *Veterinary Parasitology*.

[62] VanHoy G. (2023). *Overview of Gastrointestinal Parasites of Ruminants*.

[63] Delano M. L., Mischler S. A., Underwood W. J. (2002). Biology and Diseases of Ruminants: Sheep, Goats, and Cattle. *Laboratory Animal Medicine*.

[64] Mullen G. R., Oconnor B. M. (2019). Mites (Acari). *Medical and Veterinary Entomology*.

[65] Rojo-Vázquez F. A., Meana A., Valcárcel F., Martínez-Valladares M. (2012). Update on Trematode Infections in Sheep. *Veterinary Parasitology*.

[66] Adhikari R. B., Adhikari Dhakal M., Ghimire T. R. (2024). Intestinal Parasitism in Working Horses and Associated Zoonotic Risks in Lowlands of Nepal. *Problems of Infectious and Parasitic Diseases*.

[67] Beasley A. M., Kohn L., Ross W. (2007). *The Physiology of the Periparturient Relaxation of Immunity to Sheep Worms*.

[68] González-Garduño R., Arece-García J., Torres-Hernández G. (2021). Physiological, Immunological and Genetic Factors in the Resistance and Susceptibility to Gastrointestinal Nematodes of Sheep in the Peripartum Period: A Review. *Helminthologia*.

[69] NADIS *Parasitic Gastroenteritis (PGE) in Sheep*.

[70] Albuquerque A. C. A., Almeida F. A., Bassetto C. C., Lins J. G. G., Amarante A. F. T. (2022). Influence of Breed and Parasite Challenge on the Immune Response to Naturally Acquired Intestinal Nematode Infection in Sheep. *Journal of Helminthology*.

[71] Adhikari R. B., Adhikari Dhakal M., Ale P. B., Regmi G. R., Ghimire T. R. (2025). Prevalence of Intestinal Parasites in Captive Asian Elephants (*Elephas maximus* Linnaeus, 1758) in Central Nepal. *Veterinary medicine and science*.

[72] Tikyaa G. N., Oke P. O., Ikpa T. F., Imandeh G. N. (2019). The Effect of Water Sources, Nutritional Qualities and Management Systems on the Prevalence of Gastrointestinal Helminth Infections in Ruminants in Benue State, Nigeria. *Agro-Science*.

[73] Jia T. W., Melville S., Utzinger J., King C. H., Zhou X. N. (2012). Soil-Transmitted Helminth Reinfection After Drug Treatment: A Systematic Review and Meta-Analysis. *PLoS Neglected Tropical Diseases*.

[74] Kumar N., Rao T. K. S., Varghese A., Rathor V. S. (2013). Internal Parasite Management in Grazing Livestock. *Journal of Parasitic Diseases*.

[75] McNeily T., Wulster-Radcliffe M. (2020). *Strategies for Coping With Parasite Larvae on Pastures in the Springtime in Ohio (VME-28)*.

[76] Sissay M. M., Uggla A., Waller P. J. (2007). Epidemiology and Seasonal Dynamics of Gastrointestinal Nematode Infections of Sheep in a Semi-Arid Region of Eastern Ethiopia. *Veterinary Parasitology*.

[77] Hale M. (2006). *Managing Internal Parasites in Sheep and Goats (IP293 Slot 289 Version 050615)*.

[78] Hoarau A. O. G., Mavingui P., Lebarbenchon C. (2020). Coinfections in Wildlife: Focus on a Neglected Aspect of Infectious Disease Epidemiology. *PLoS Pathogens*.

[79] Adhikari R. B., Ghimire T. R. (2021). A Case Study of Multiple Parasitisms in a Calf Buffalo (*Bubalus Bubalis*). *ASD*.

[80] Gillan N. (2016). *Yersinia Pseudotuberculosis Enteritis in Adult Sheep*.

[81] NSW (2019). *Case Study—From Flash to Flat*.

[82] Mabbott N. A. (2018). The Influence of Parasite Infections on Host Immunity to Co-Infection With Other Pathogens. *Frontiers in Immunology*.

[83] Sweeny A. R., Corripio-Miyar Y., Bal X. (2022). Longitudinal Dynamics of Co-Infecting Gastrointestinal Parasites in a Wild Sheep Population. *Parasitology*.

[84] Vagenas D., Bishop S., Kyriazakis I. (2007). A Model to Account for the Consequences of Host Nutrition on the Outcome of Gastrointestinal Parasitism in Sheep: Logic and Concepts. *Parasitology*.

