# Supplementary material for: Prevalence of Coccidia and Other Intestinal Parasites in Indigenous Sheep (Ovis aries) in an Agricultural Area in Central Nepal
Source: Vet Med Int. 2025 Jun 20;2025:1033918. doi: 10.1155/vmi/1033918 (PMC12204748; doi:10.1155/vmi/1033918)
Supplement: Supporting Information — Additional supporting information can be found online in the Supporting Information section. [file 1033918.f1.docx]

| 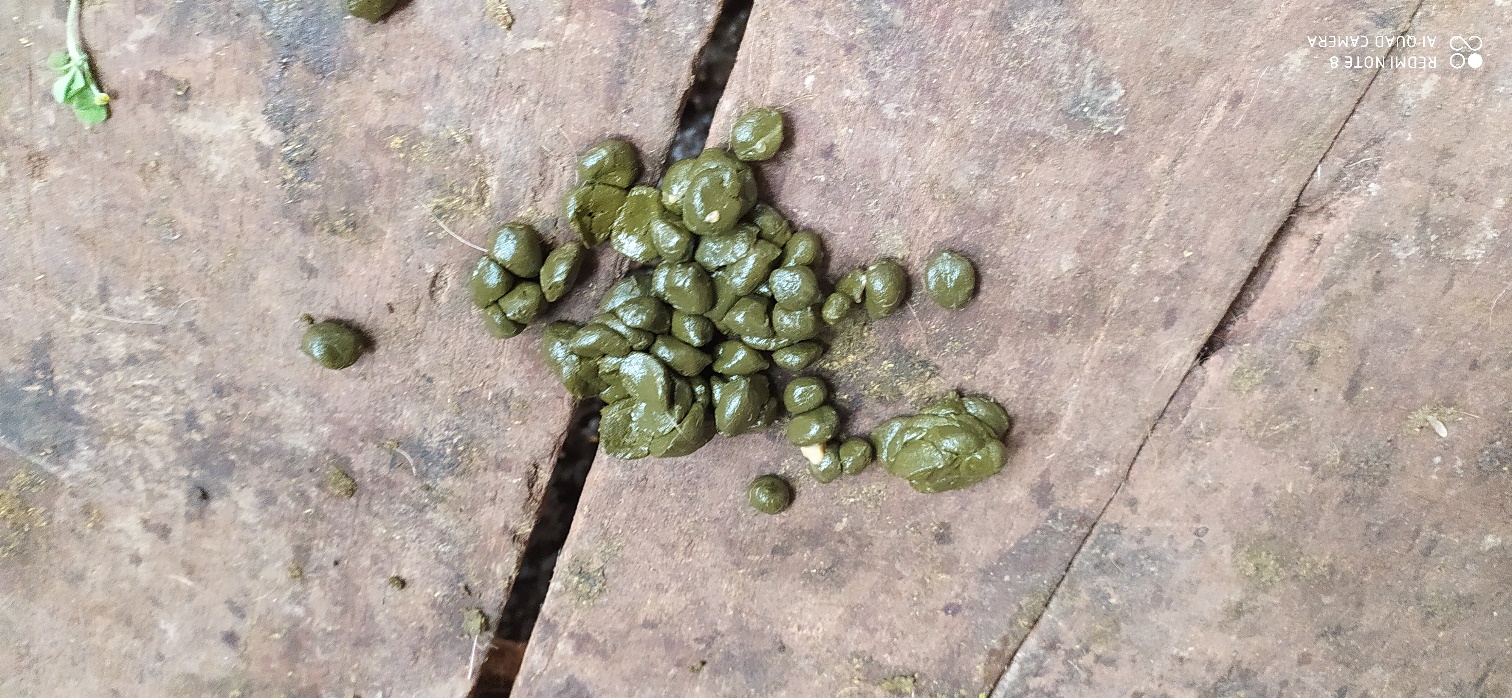 | 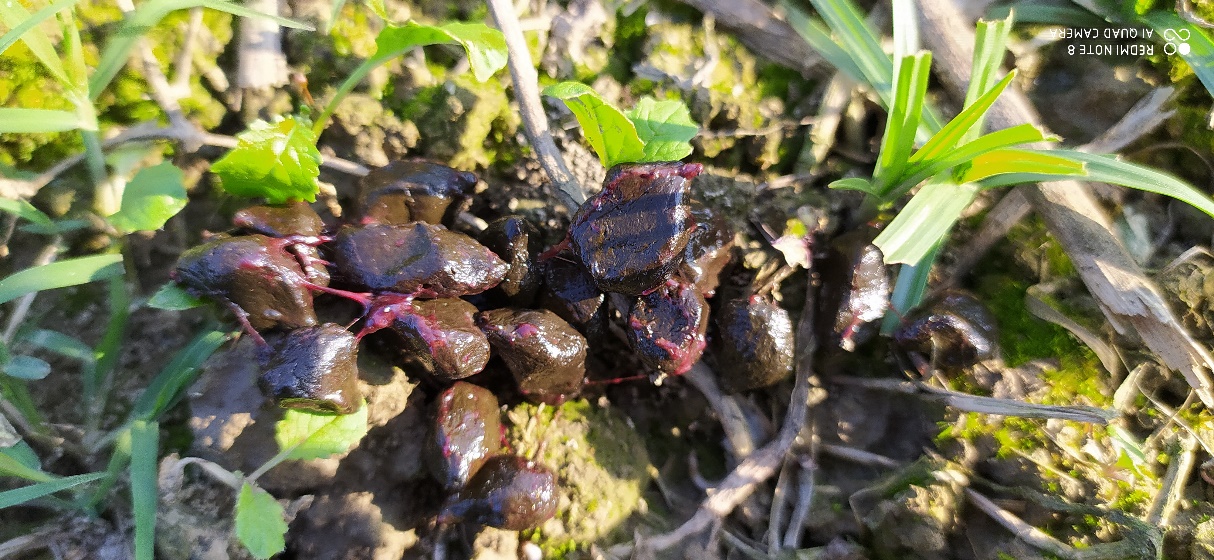 |
| --- | --- |
| **a) Soft stool** | **b) Constipated stool with bloody mucus** |
| 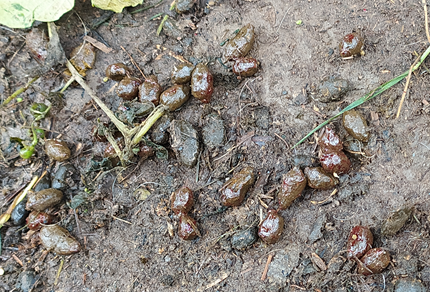 | 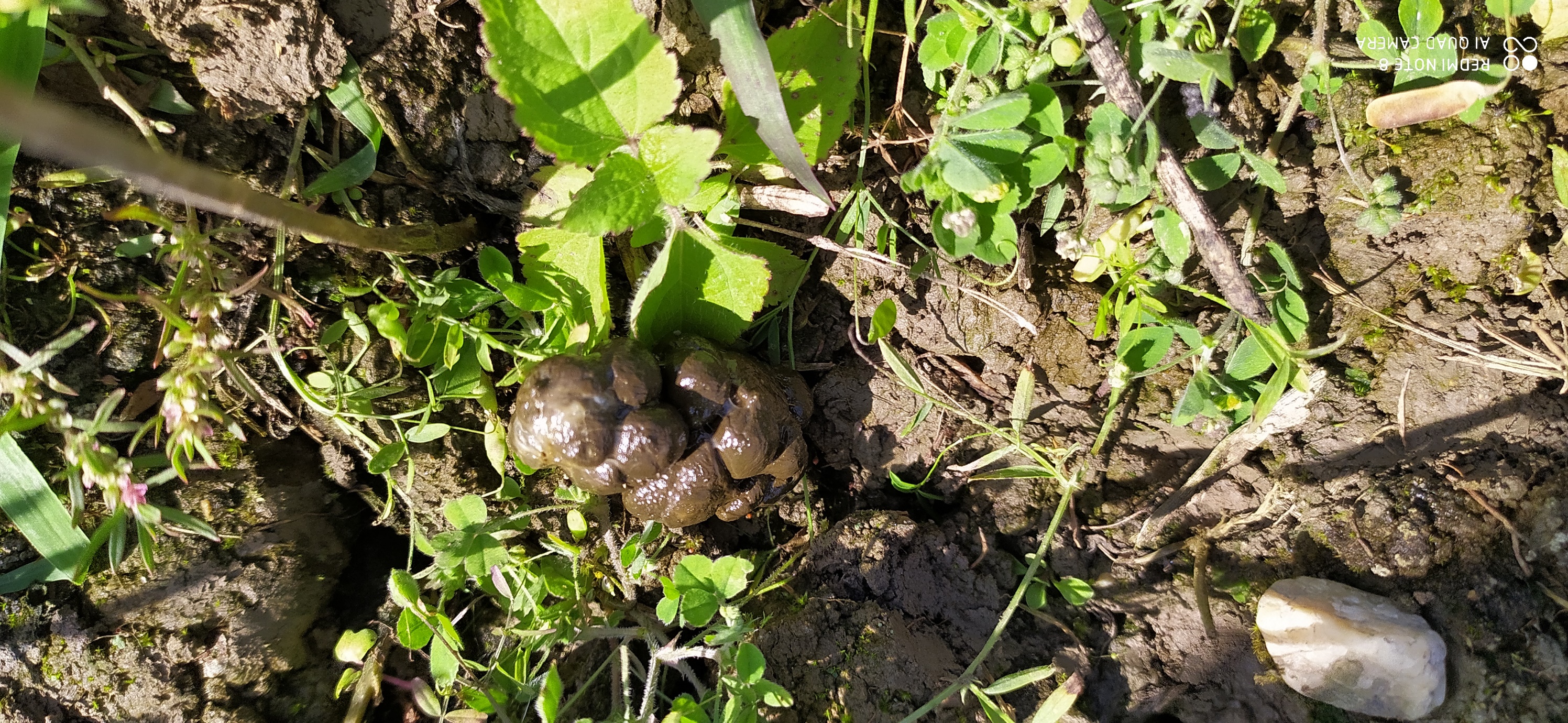 |
| **c) Bloody stool** | **d) Mucus-coated stool** |
| 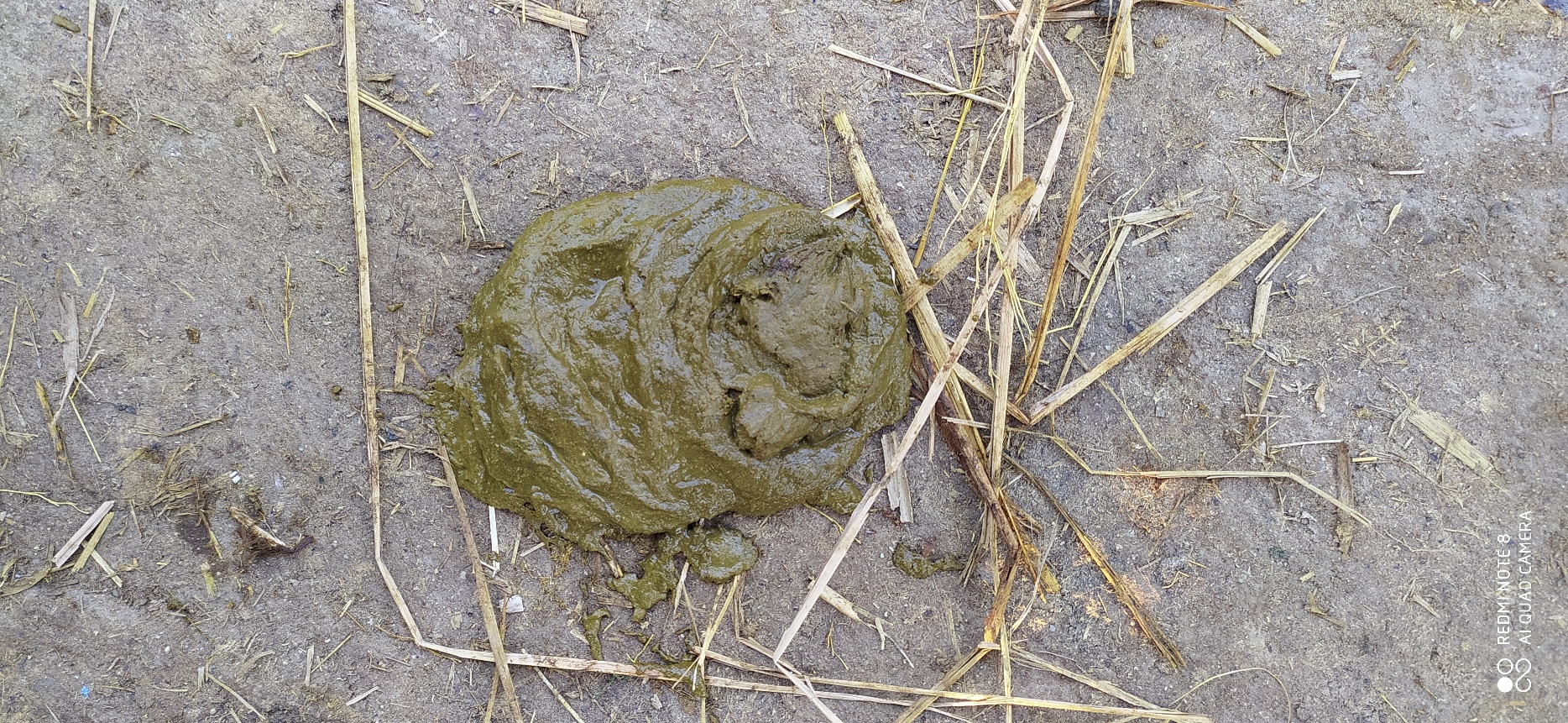 | 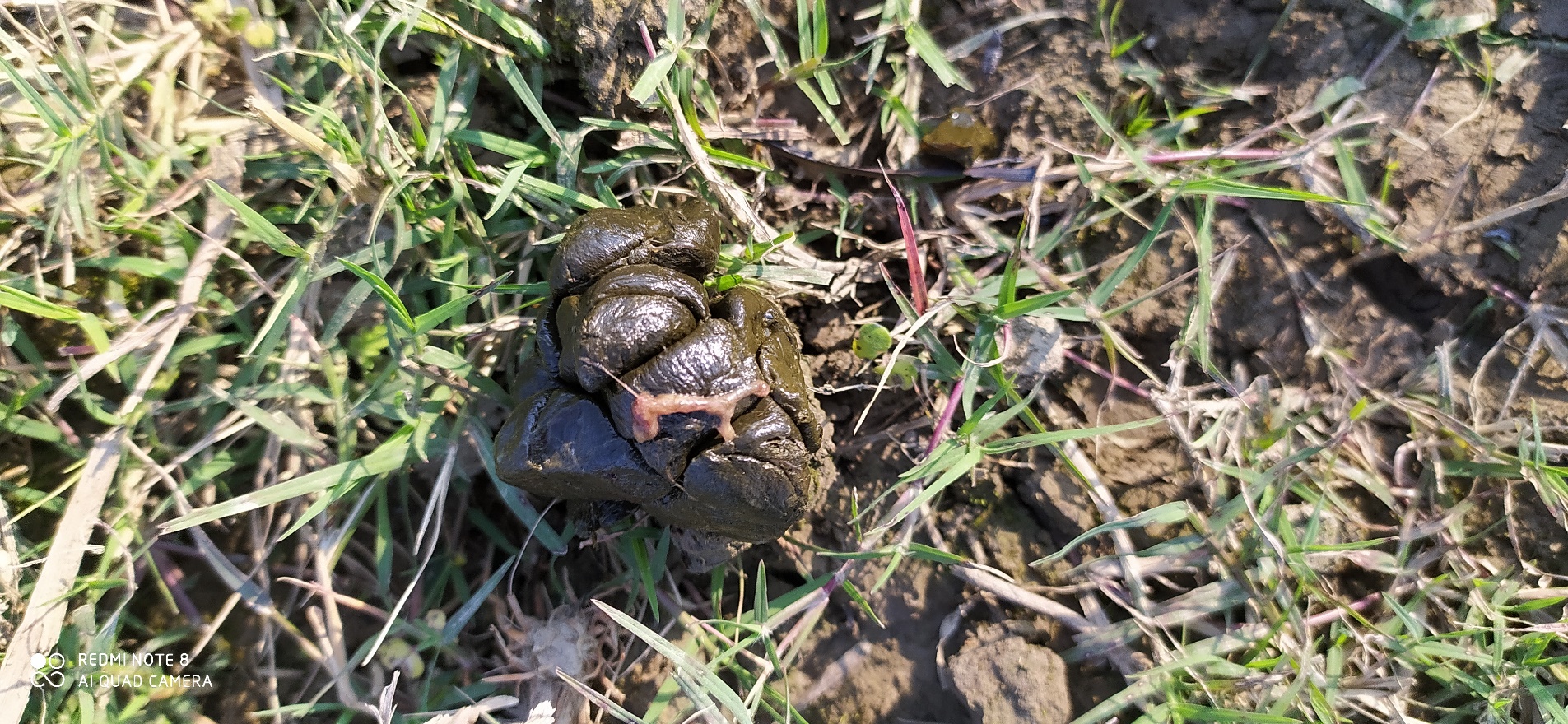 |
| **e) Diarrheal stool** | **f) Mucoid stool** |
| 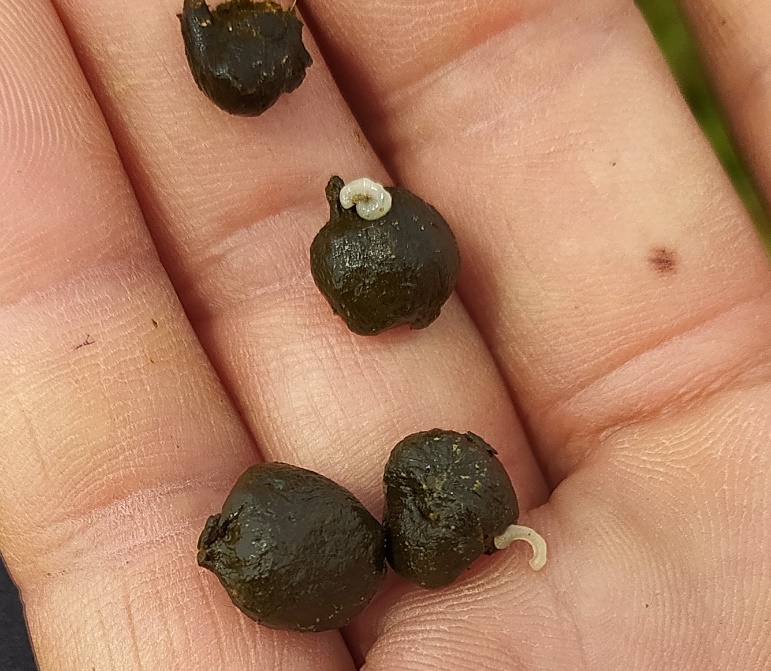 |  |
| **g) Stool with gravid segments of tapeworms** |  |

**Supplementary Figure 1:** Fecal consistency of indigenous sheep

**Supplementary Table 1:** Oocyst characteristics of *Eimeria* spp. in indigenous sheep (n= number of oocyst/sporozoite measured)

| ***Eimeria* spp.** | **Oocyst Characters** | | | | | **Sporozoites** | |
| --- | --- | --- | --- | --- | --- | --- | --- |
|  | **Micropyle** | **Shape of oocyst** | **Oocyst wall** | **Length x breadth (µm)** | **Shape index (l/b)** | **Length x breadth (µm)** | **Shape index l/b** |
| *Eimeria pallida* | Absent | Rounded or slightly oval | Smooth | 12-22 (19.5) x 12-19  (16) | 1.2  n=20 | 7-10 (8.4) x 4-6 (5) | 1.9  n=10 |
| *E. parva* | Absent | Oval-shaped | Smooth | 19-25 (22.7) x 15-23 (18.9) | 1.2  n=15 | 8-11 (9.3) x 5-6 (5.4) | 1.7  n=16 |
| *E. marsica* | Present | Elongated oval shape | Smooth | 22-30 (26.5) x 15-20 (17.8) | 1.5  n=10 | 7-11 (9.9) x 6-10 (6.3) | 1.6  n=15 |
| *E. ovinoidalis* | Absent | Oval | Smooth | 22-30 (26.2) x 18-25 (20.7) | 1.3  n=15 | 9-12 (10.8) x 6-8 (6.4) | 1.7  n=10 |
| *E. webridgensis* | Present | Oval, slightly elongated | Smooth | 25-28 (26.5) x (17-20)  (19.4) | 1.4  n=10 | 9-14 (10.7) x 5-8 (5.7) | 1.9  n=12 |
| *E. crandallis* | Present | Oval with a somewhat tapered end | Smooth | 24-32 (29.2) x 17-21 (19.3) | 1.5  n=12 | 8-11 (9.5) x 6-8 (7) | 1.4  n=12 |
| *E. faurei* | Absent | Oval with a slightly tapered end | Smooth | 26-34 (31.6) x (22-26)  (23.2) | 1.4  n=10 | 9-16 (11.7) x 7-9 (7.7) | 1.5  n=10 |
| *E. granulosa* | Present | Oval with terminating non-micropylar end | Smooth | 33-36 (35) x 19-25 (21.3) | 1.6  n=10 | 10-12 (11.2) x 6-8 (6.7) | 1.7  n=10 |
| *E. bakuensis* | Present | Oval/elongated. | Smooth | 32-41 (35.9) x 20-28  (23.9) | 1.5  n=15 | 9-14 (11.4) x 6-8 (6.9) | 1.7  n=16 |
| *E. ahsata* | Present | Oval, with slightly narrowing non-micropylar end | Smooth | 34-44 (37.2) x 21-29 (25.4) | 1.5  n=30 | 11-18 (15.3) x 6-11 (7.9) | 1.9  n=21 |
| *E. intricate* | Present | Large, round-to-oval shape | slightly rough and highly thickened | 42-56 (49.6) x 31-38  (35.5) | 1.4  n=15 | 14-16 (14.9) x 10-13 (11.4) | 1.3  n=12 |

**Supplementary Table 2:** OPG/EPG of coccidian and nematode parasites in the fecal samples concerning the age of the sheep

| **Parasites** | **Average range of OPG/EPG** | | |
| --- | --- | --- | --- |
|  | **Lambs (<1Year)** | **Hogget (>1-<2 Years)** | **Adult (≥2-10Years)** |
| ***Eimeria* spp.** | 400–13,500 | 300–12,500 | 200–7200 |
| ***Moniezia* spp.** | 400–1600 | 300–2,400 | 400–3,200 |
| **Ascarid sp.** | 100–400 | - | - |
| **Strongyle** | 200-5,500 | 200-8,000 | 200–9,500 |
| ***Trichuris* *ovis*** | 100–800 | 100–1200 | 100–1,000 |
| ***Strongyloides* sp.** | 100–1400 | 100–1200 | 100–1,000 |
| ***Capillaria* sp.** | - | 200 | 100–600 |
